# Declining antibody levels to *Trypanosoma cruzi* correlate with polymerase chain reaction positivity and electrocardiographic changes in a retrospective cohort of untreated Brazilian blood donors

**DOI:** 10.1371/journal.pntd.0008787

**Published:** 2020-10-27

**Authors:** Lewis F. Buss, Léa Campos de Oliveira- da Silva, Carlos H. V. Moreira, Erika R. Manuli, Flavia C. Sales, Ingra Morales, Clara Di Germanio, Cesar de Almeida-Neto, Sonia Bakkour, Paul Constable, Marcelo M. Pinto-Filho, Antonio L. Ribeiro, Michael Busch, Ester C. Sabino

**Affiliations:** 1 Instituto de Medicina Tropical da Faculdade de Medicina (FMUSP) da Universidade de São Paulo, São Paulo, Brazil; 2 Vitalant Research Institute, San Francisco, CA, United States of America; 3 Fundação Pró-Sangue—Hemocentro de São Paulo, São Paulo, Brazil; 4 Ortho Clinical Diagnostics, Rochester, NY, United States of America; 5 Telehealth Center, Hospital das Clínicas, and Internal Medicine Department, School of Medicine, Universidade Federal de Minas Gerais, Belo Horizonte, Brazil; 6 Department of Laboratory Medicine, University of California San Francisco, San Francisco, CA, United States of America; Universidad de Buenos Aires, ARGENTINA

## Abstract

**Background:**

Although infection with *Trypanosoma cruzi* is thought to be lifelong, less than half of those infected develop cardiomyopathy, suggesting greater parasite control or even clearance. Antibody levels appear to correlate with *T*. *cruzi* (antigen) load. We test the association between a downwards antibody trajectory, PCR positivity and ECG alterations in untreated individuals with Chagas disease.

**Methodology/Principal findings:**

This is a retrospective cohort of *T*. *cruzi* seropositive blood donors. Paired blood samples (index donation and follow-up) were tested using the VITROS Immunodiagnostic Products Anti-*T*.*cruzi* (Chagas) assay (Ortho Clinical Diagnostics, Raritan NJ) and PCR performed on the follow-up sample. A 12-lead resting ECG was performed. Significant antibody decline was defined as a reduction of > 1 signal-to-cutoff (S/CO) unit on the VITROS assay. Follow-up S/CO of < 4 was defined as borderline/low. 276 untreated seropositive blood donors were included. The median (IQR) follow-up was 12.7 years (8.5–16.9). 56 (22.1%) subjects had a significant antibody decline and 35 (12.7%) had a low/borderline follow-up result. PCR positivity was lower in the falling (26.8% vs 52.8%, p = 0.001) and low/borderline (17.1% vs 51.9%, p < 0.001) antibody groups, as was the rate of ECG abnormalities. Falling and low/borderline antibody groups were predominantly composed of individuals with negative PCR and normal ECG findings: 64% and 71%, respectively.

**Conclusions/Significance:**

Low and falling antibody levels define a phenotype of possible spontaneous parasite clearance.

## Author summary

Infection with the single-celled parasite *Trypanosoma cruzi* (Chagas disease) is thought to be lifelong. However, only a third of infected people develop Chagas cardiomyopathy–the main disease manifestation. This may reflect the different extent to which individuals control the parasite, with some potentially clearing it entirely. In chronically infected immunocompetent patients, a marker of parasite burden is the quantity of antibody against *T*. *cruzi* in the blood: more parasite, more immune stimulation, more antibody. In this study we show how antibody levels change over many years in a cohort of untreated patients with Chagas disease. We find that among individuals with falling or low/borderline antibody levels there was a lower rate of parasite detection in the blood and a lower rate of cardiomyopathy. 60% of subjects with falling antibody levels had no evidence of active disease, twice as many as among patients with other antibody trajectories (stable or rising). Our findings support an account of the natural history of Chagas disease in which a proportion of those infected achieve a greater control of the parasite, with some individuals potentially clearing it completely.

## Introduction

Chagas disease (CD)–due to infection by *Trypanosoma cruzi*–is a neglected tropical disease. It affects 6–8 million people worldwide [1] and is responsible for 50,000 deaths annually [2,3]. Vectoral transmission is by contact with the feces of the infected triatomine bug, with approximately 70 million people living in areas at risk of exposure [4]. Acute infection is characterized by a mild illness in which the parasite is readily detected in the blood. This gives way to chronic tissue sequestration, particularly affecting the myocardium. However, the development of clinically apparent cardiomyopathy is variable, with most individuals remaining asymptomatic for life [5]. The mechanisms underlying this process remain poorly understood.

One hurdle to understanding the natural history Chagasic cardiomyopathy is a lack of reliable biomarkers of parasite persistence. Parasitaemia in the chronic phase is both low-grade and intermittent, and consequently diagnosis is based on serology [6,7]. When asymptomatic individuals are systematically screened–such as is the case for blood donors–a third of those identified as seropositive have low antibody levels or discordant test results[8]. These borderline cases show spatial clustering [9] and shared risk factors [8,10,11] with unequivocal seropositive cases. This suggests there was a true exposure to *T*. *cruzi*, as opposed to a simple false-positive result.

One explanation for these findings is that a proportion of infected individuals may achieve control of the parasite or, in some cases, completely clear it [12–14]. This would reduce, or completely remove, the antigenic stimulus, leading to slow seroreversion. Indeed, antibody levels have been shown to correlate with peripheral parasitaemia in a cross-sectional blood donor cohort [15]. In that same cohort, antibody trend overtime was measured in a subset of donors with more than one sample time point. The inclusion criteria (positive results on three relatively low-sensitivity assays) led to a selection bias for donors with high antibody levels at index donation. Six donors had a significant decline in antibody level and all were PCR-negative. As such, further data are needed to properly assess the existence of spontaneous seroreversion and its association with parasitaemia. Furthermore, the relationship between antibody level and clinically apparent disease–namely Chagasic cardiomyopathy–has not been reported.

Herein we characterize the trajectory of antibody levels over a number of years in a group of untreated Brazilian seropositive blood donors. We test the hypothesis that individuals with a downwards antibody trajectory have controlled (or potentially cleared) the parasite–as indicated by a negative polymerase chain reaction (PCR) and absence of typical electrocardiographic abnormalities.

## Methods

### Study design

This is a retrospective cohort of *T*. *cruzi* seropositive blood donors identified in routine blood donation screening at the *Fundação Pró-Sangue* blood bank in São Paulo, Brazil. Blood donations were made between 1996 and 2015. The screening tests varied over this time period due to the availability and cost (see S1 Table). Eligible seropositive donors were identified in institutional records and invited to attend an enrolment appointment in 2017–2018. At this appointment participants answered a questionnaire about Chagas exposure and cardiovascular risk factors. Those that reported previous benznidazole treatment were excluded. At this time a blood sample was taken for *T*. *cruzi* polymerase chain reaction (PCR) and serology testing. All participants underwent a standard resting 12-lead electrocardiogram (ECG).

### VITROS Immunodiagnostic Products Anti-*T*.*cruzi* (Chagas) assay

Original blood donation samples were retrieved from storage and an aliquot taken for processing. The donation samples (1996–2015) and the samples provided at cohort enrolment (2017–2018) were sent to the Vitalant Research Institute (San Francisco, CA) for analysis using the VITROS Immunodiagnostic Products Anti-*T*.*cruzi* (Chagas) assay (Ortho Clinical Diagnostics, Raritan NJ). The protocol was performed according to the manufacturer’s instructions. The test is an enzyme-linked immunosorbent assay (ELISA) that utilizes MicroWells coated with a whole cell lysate containing *T*.*cruzi* antigens as the solid phase with chemiluminescent detection. Results are reported as the ratio of signal-to-cutoff (S/CO) which is a function of the amount of anti-*T*. *cruzi* antibody present in the test sample. The sensitivity and specificity are 97.7% and 100%, respectively [16].

We categorized the VITROS Anti-*T*. *cruzi* test results as low- and high-positive using a S/CO value of four as the threshold. This value was shown to be the nadir of a bimodal distribution of VITROS Anti-*T*. *cruzi* results in an endemic area of Argentina [8]. As such, it may distinguish patients with resolving or progressive disease trajectories. Furthermore, the difference between S/CO at follow-up and donation was calculated such that negative values indicate a falling S/CO. A decrease in S/CO of greater than one unit was used to define a subpopulation with significantly decreasing antibodies [15].

### Polymerase chain reaction (PCR)

Blood samples collected at the study enrollment visit (2017–2018) were tested using a target-capture (TC) real-time (RT) PCR assay, as previously described [17]. Capture of *T*. *cruzi* was performed using magnetic beads coated with the three 20-mer capture oligonucleotides:

TCZ 1 CGAGCTCTTGCCCACACGGGAAAAAAAAAAAAAAAAAAAAAAAAAA;

TCZ 2 CCTCCAAGCAGCGGATAGTTCAGGAAAAAAAAAAAAAAAAAAAAAA AAAA and; TCZ 3 TGCTGCASTCGGCTGATCGTTTTCGAAAAAAAAAAAAAAAAAAAAA AAAAAA.

The captured DNA targets were eluted from the magnetic beads and amplified on Applied Biosystems 7500. Briefly, 25uL of DNA was added to 50uL of BSRI PCR mix. The PCR conditions were 10min at 95°C, followed by 45 cycles of 30 sec at 95°C, 30 sec at 64°C, 45 sec at 72°C. After completion of thermal cycling a dissociation step was performed, and the melting curve was analyzed. Product dissociations with one or two peaks at 80–82°C were considered positive if the cycle threshold (CT) was less than 45 cycles. Eight replicate assays were performed and the result was considered positive if at least two replicates produced a specific products based on dissociation analysis.

### Electrocardiogram

ECGs were analyzed using an automated system with manual overreading and classification according to the Minnesota code. Major and minor electrocardiographic alterations were defined as previously described [18]. The final ECG classification was “major” if the ECG had any major alterations, “minor” if it had only minor alterations, and “normal” if it had neither minor nor major alterations. Furthermore, a subset of ECG findings was defined as typical of Chagas disease [19] following the 2^nd^ Brazilian consensus on Chagas disease (2015) [20]–see S2 Table.

### Statistical analysis

The exposures of interest were the change in VITROS S/CO between donation and follow-up timepoints, and the absolute S/CO value at both these time points separately. The outcome variables were PCR status and ECG alterations at follow-up. We treated the outcome variables in the analysis as follows. Firstly, PCR status was considered in isolation: positive versus negative. Secondly, the ECG was analysed in isolation, considering three different aspects: the presence versus absence of individual ECG alterations; the final ECG classification (major, minor, or normal); and the total number of typical Chagas disease alterations per trace (0, 1, or 2+). Finally, we made a joint PCR and ECG endpoint consisting of participants with both a negative PCR and an ECG free of major alterations (i.e. normal or minor alterations only). This joint endpoint provides the strongest evidence of disease inactivity, as neither parasitaemia nor cardiac involvement were identified.

Univariate analyses comparing PCR and ECG variables between categorical antibody groups were performed with the Chi-squared or Fisher exact test. Furthermore, the continuous S/CO distributions (donation, follow-up, and follow-up–donation) were compared according to the PCR/ECG joint endpoint with the Wilcoxon rank sum test.

Finally, we sought to assess the independent association between antibody level (donation, follow-up and follow-up–donation) and the joint PCR/ECG endpoint. We built multiple logistic regression models with the joint PCR/ECG end point as the dependent variable. We tested age, sex, comorbidities and smoking status as potential confounding variables in the models. Variables were retained in the model if they altered the odds ratio (OR) associated with antibody level by > 5%.

### Ethics statement

The study protocol was submitted and approved by the local Ethics committee at Hospital of Clinics–Fundacão Pró-Sangue and CAPESPQ, University of São Paulo (CEP FMUSP 1.604.712). All participants provided written informed consent before enrolment.

## Results

### Cohort characteristics

The initial cohort consisted of 279 untreated seropositive blood donors. Three false-positive cases (non-reactive VITROS Anti-*T*. *cruzi* results at both time points and negative PCR at follow-up) were excluded, resulting in 276 cases for analysis. Twenty-three donation samples were insufficient to run the VITROS Anti-*T*. *cruzi* assay. Therefore, analyses involving only the follow-up sample consisted of 276 participants and analyses involving the donation sample consisted of 253 participants. Subject characteristics are presented in Table 1.

**Table 1:** Characteristics of 276 *T*. *cruzi*-seropositive blood donors responding to the follow-up questionnaire (2017–2018).

Subject characteristics	Full cohort N = 276 n (%) or median (IQR)
**Age (years)**	
< 40	15 (5.5)
40–49	56 (20.4)
50–59	96 (35.0)
60–69	82 (29.9)
> = 70	25 (9.1)
**Sex**	
Male	133 (48.2)
Female	143 (51.8)
**Country of origin**	
Brazil	274 (99.3)
Argentina	1 (<1)
Bolivia	1 (<1)
**Self-reported skin color**	
White	128 (46.4)
Black	39 (14.1)
Mixed (*pardo*)	98 (35.5)
Other	11 (4.0)
**Educational level**	
No schooling	10 (3.6)
Incomplete primary schooling	102 (37.0)
Complete primary schooling	76 (27.5)
Complete secondary schooling	67 (24.3)
College, technical or above	21 (7.6)
**Comorbidities**	
Diabetes	34 (12.6)
Renal disease	12 (4.5)
Stroke	7 (2.5)
Myocardial infarction	9 (3.3)
Hypertension	103 (38.0)
High cholesterol	94 (36.6)
**Smoking status**	
Current smoker	24 (8.7)
Ex-smoker	108 (39.1)
Never smoker	144 (52.2)
Follow-up* (years)	12.7 (8.5–16.9)

* time (in years) between the original donation date and follow-up visit. PCR–polymerase chain reaction; ECG–electrocardiogram; IQR–interquartile range. Missing data: 6 diabetes; 8 renal disease; 1 stroke; 1 myocardial infarction; 5 hypertension; 19 high cholesterol

### VITROS Immunodiagnostic Products Anti-*T*.*cruzi* (Chagas) assay

**Fig 1:**
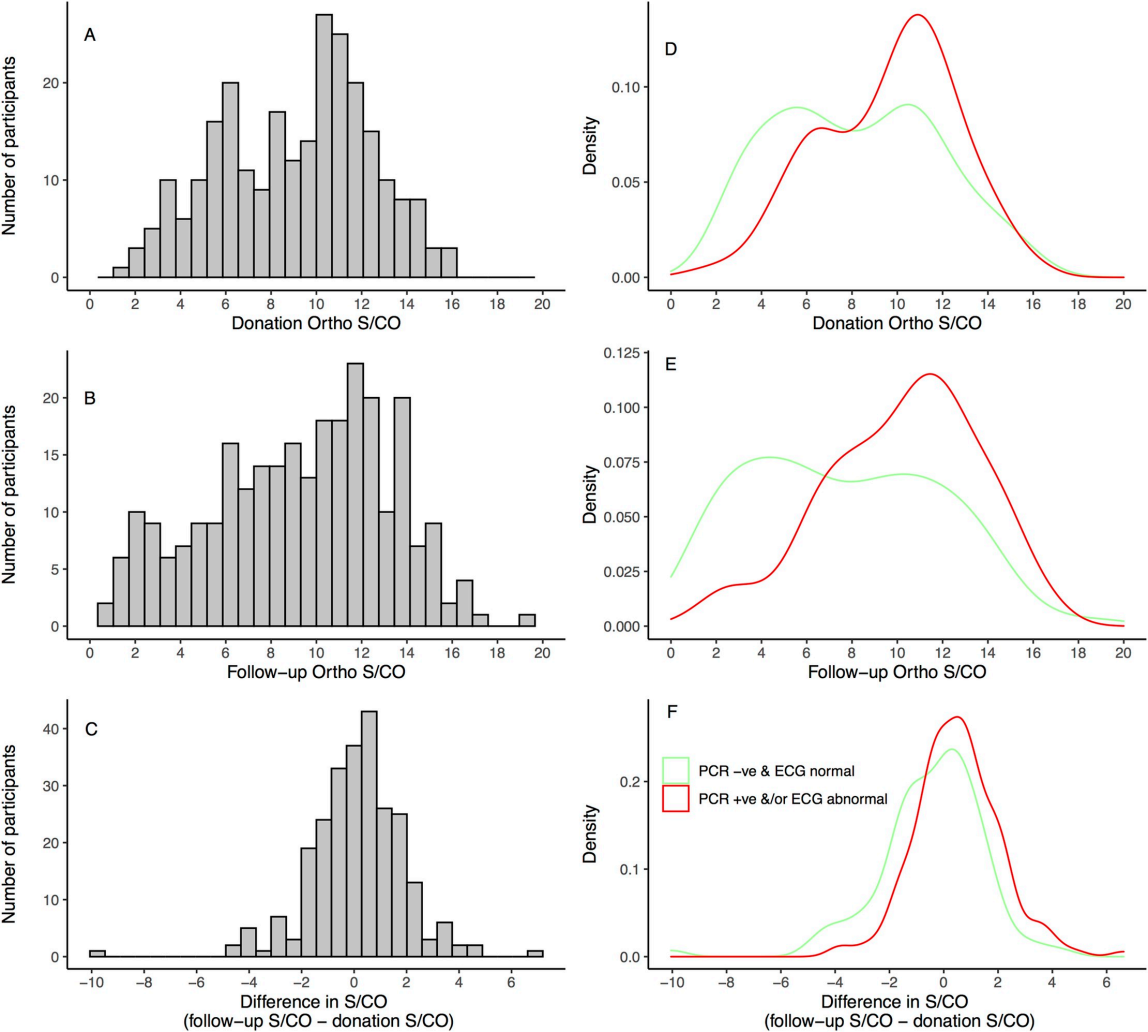
Histograms of the VITROS Anti-*T*. *cruzi* signal-to-cutoff (S/CO) values among participating blood donors at donation (A) and follow-up (B), and the distribution of the difference in S/CO between the two time points (C). Smoothed kernel density plots of the distributions of donation (D), follow-up (E) and the change in S/CO (F) stratified according to PCR and ECG result (PCR -ve & ECG normal versus PCR+ve &/or ECG abnormal). In panel D, the median [IQR] S/CO in the PCR +ve &/or ECG abnormal donors is 10.2 [7.2 to 11.7], and among PCR-ve & ECG normal donors is 8.2 [5.3 to 10.9], p = 0.001 (Wilcoxon rank sum). In Panel E, comparing the same groups, the median [IQR] values are 10.6 [7.9 to 12.6] versus 7.0 [4.2 to 11.1], respectively, p <0.001 (Wilcoxon rank sum). In panel F, the median [IQR] values are respectively 0.46 [-0.4 to 1.4] and -0.2 [-1.4 to 0.76], p<0.001 (Wilcoxon rank sum).

The median (IQR) time period between blood donation and the follow-up appointment was 12.7 years (8.5–16.9). The distributions of S/CO values at donation and follow-up are presented in Fig 1A and Fig 1B. There is a bimodal distribution of ELISA S/CO values in the donation samples. At follow-up the S/CO values are more widely spread and may also represent the superimposition of two distinct distributions, although this is less clear. The change in S/CO between the two time points is shown in Fig 1C and follows a roughly normal distribution.

**Fig 2:**
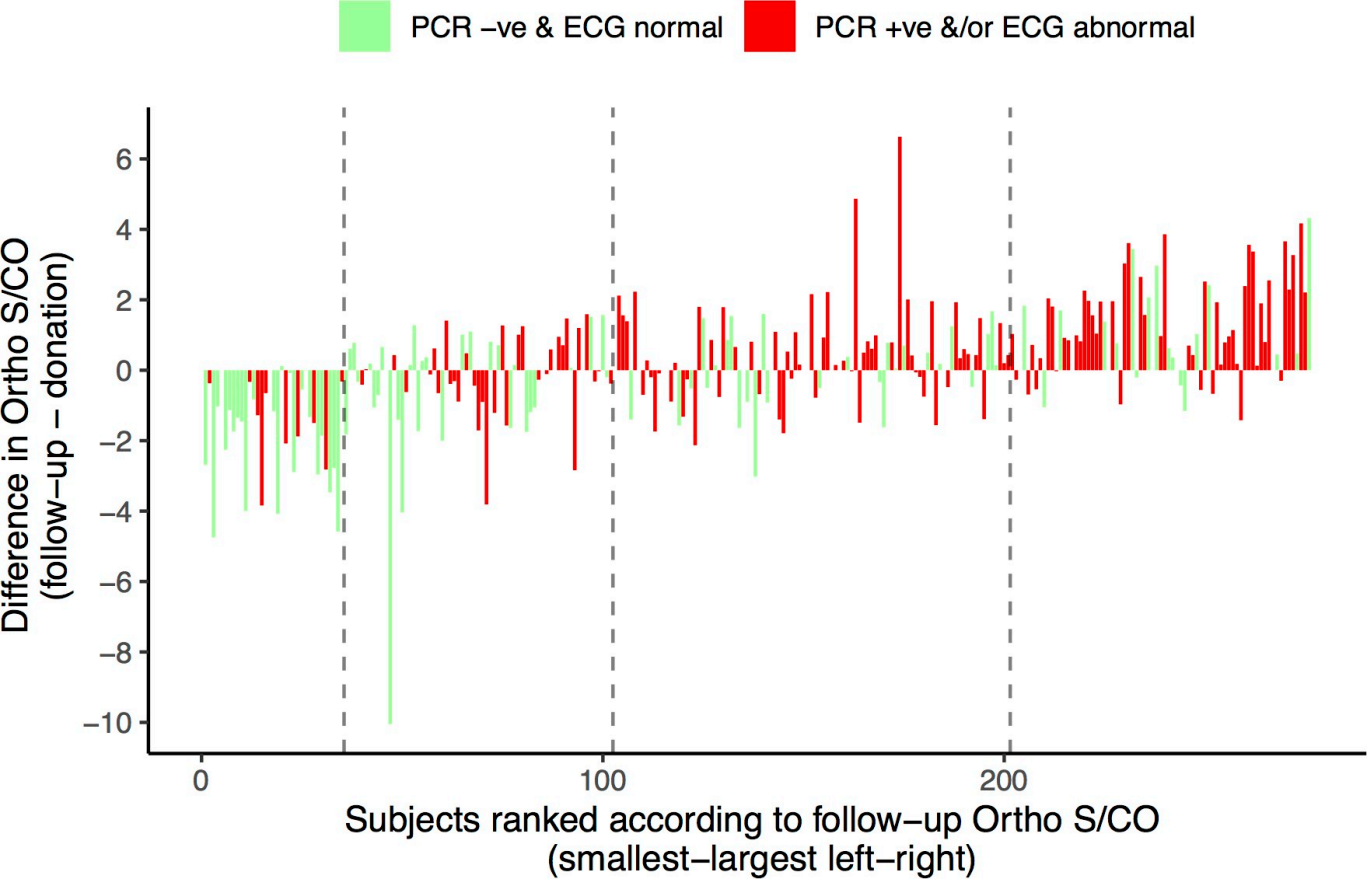
Waterfall plot of the change in S/CO between donation and follow-up visits for 253 subjects with results available for both time points. Subjects with negative PCR and an ECG free of major alterations (PCR -ve & ECG normal) are represented with green bars, whereas subjects with a positive PCR and/or ECG with a major alterations (PCR +ve &/or ECG abnormal) are shown in red. Subjects are ranked (horizontal axis) according to the final VITROS Anti-*T*. *cruzi* ELISA S/CO, such that subjects with the lowest S/CO values at follow-up are shown on the far left, and those with the highest follow-up S/CO values are shown on the far right. Vertical dashed lines mark the rank positions corresponding to S/CO values of 4, 8 and 12 (left to right)–e.g. subjects to the left of the first dashed in had follow-up S/CO values < 4.

Fig 2 presents the individual changes in S/CO between donation and follow-up for all 253 subjects with both timepoints available. Subjects are ordered according to their follow-up S/CO value. It is apparent from this figure that donors with lower S/CO values at follow-up in general underwent a decline in S/CO compared to their donation sample. By contrast, those with higher follow-up S/CO values in general underwent an increase in S/CO compared to the donation value.

Fifty-six (22.1%) subjects had a significant (> 1 S/CO unit) decline in ELISA reactivity and two participants became seronegative (S/CO < 1) at follow-up. The first fell from a S/CO of 1.2 to 0.78 over the course of 5 years. The second fell from a S/CO of 3.3 to 0.56 over 17 years. The S/CO was unchanged (varied by < 1 S/CO between donation and follow-up) in 126 (50%) donors and 71 (28%) underwent an increase of >1 S/CO unit (see S3 Table). Thirty-five (12.7%) donors had a follow-up S/CO of < 4 and 241 (87.3%) of ≥ 4; on the donation sample 19 (7.5%) and 234 (92.5) were < 4 or ≥ 4 respectively. Demographic characteristics and cardiovascular risk factors were similar across the groups defined by ELISA S/CO (S4 Table).

### Univariate associations between ELISA S/CO, PCR result and ECG alterations

One hundred and thirty-one (47.5% of 276) subjects had a positive PCR result on the follow-up sample. In donors with S/CO values that fell by more than one S/CO unit, the PCR positivity rate was lower than the rest of the cohort (26.8% vs 52.8%—see Table 2). Further stratifying subjects into stable antibody level (increase/decrease < 1 S/CO) and increasing antibody levels (increase > 1 S/CO) the proportion with positive PCR was similar: 52.4% and 53.5%, respectively (see S3 Table). There was a lower PCR positivity among the donors with low (<4) compared to high (≥ 4) S/CO values on both follow-up and donation samples (Table 2).

**Table 2:** PCR result and ECG findings at the follow-up visit among *T*. *cruzi-*seropositive blood donors.

Disease parameters	Reduction in S/CO > 1 n = 56	Reduction of S/CO < 1 or increase n = 197	p-value	Follow-up S/CO < 4 n = 35	Follow-up S/CO ≥ 4 n = 241	p-value	Donation S/CO < 4 n = 19	Donation S/CO ≥ 4 n = 234	p-value
**PCR result**									
Positive	15 (26.8)	104 (52.8)		6 (17.1)	125 (51.9)		3 (15.8)	116 (49.6)	
Negative	41 (73.2)	93 (47.2)	0.001	29 (82.9)	116 (48.1)	<0.001	16 (84.2)	118 (50.4)	0.009
**Final ECG classification**, n (%)									
Major	11 (19.6)	76 (38.6)		4 (11.4)	94 (39.0)		1 (5.3)	86 (36.8)	
Minor	29 (51.8)	82 (41.6)		19 (54.3)	101 (41.9)		12 (63.2)	99 (42.3)	
Normal	16 (28.6)	39 (19.7)	0.028	12 (34.3)	46 (19.1)	0.004	6 (31.6)	49 (20.9)	0.010
**Typical changes**, n (%)									
0	46 (82.1)	122 (61.9)		31 (88.6)	149 (61.8)		18 (94.7)	150 (64.1)	
1	6 (10.7)	57 (28.9)		3 (8.6)	67 (27.8)		0 (0)	63 (26.9)	
2+	4 (7.1)	18 (9.1)	0.010	1 (2.9)	25 (10.4)	0.006	1 (5.3)	21 (9.0)	0.009
**Joint PCR/ECG endpoint,** n(%)									
PCR-ve & ECG normal	36 (64.3)	62 (31.5)		25 (71.4)	80 (33.2)		15 (78.9)	83 (35.5)	
PCR+ve &/or ECG abnormal	20 (35.7)	135 (68.5)	<0.001	10 (28.6)	161 (66.8)	<0.001	4 (21.1)	151 (64.5)	<0.001

PCR–polymerase chain reaction; ECG–electrocardiogram. Final ECG classification: major if at least one major alteration, minor if only minor alterations present, normal if neither minor nor major alterations present. Typical ECG changes refer to alterations typical of Chagas cardiomyopathy following the 2^nd^ Brazilian consensus on Chagas disease. P-values are calculated by Chi-squared test or Wilcoxon rank sum, as appropriate.

The proportion of donors with individual major and typical Chagas disease ECG alterations is shown according to antibody level in S2 Table. Of note, right bundle branch block (RBBB) was half as frequent in donors with falling antibody levels compared to those with stable or increasing levels (10.7% vs 20.3%, p = 0.14). This difference was more pronounced in donors with low (<4) versus high (≥ 4) S/CO levels: 2.9% (S/CO < 4) versus 19.9% (S/CO ≥ 4) with RBBB (p = 0.026), and 0.0% (S/CO < 4) versus 19.7% (S/CO ≥ 4) with RBBB (p = 0.029), at follow-up and donation timepoints, respectively. The proportion of ECGs classified as having a major alteration, and the number of typical Chagas alterations per trace, were lower in the decreasing antibody group, as well as the S/CO < 4 groups (Table 2). Donors with increasing antibody levels (increase > 1 S/CO) had the highest number of typical alterations per trace (S3 Table).

Considering the joint PCR/ECG end point, donors with negative PCR and normal ECG were more frequent in the falling antibody group, as well as the S/CO <4 groups at donation and follow-up (Table 2). We observe bimodal distributions of S/CO values at donation and follow-up in the PCR-ve & ECG normal group (Fig 1D and 1E). Fig 2 shows both the change in S/CO and the follow-up value for all participants. Notably, the PCR-ve and ECG normal cases cluster among donors with low and falling antibody levels.

### Multivariate associations between ELISA S/CO and the joint PCR/ECG endpoint

We assessed the effect of age, sex, comorbidities (hypertension and diabetes) and smoking status as potential confounding variables for the relationship between antibody level and the joint PCR and ECG endpoint. Table 3 shows the results of the univariable logistic regression models for these variables. Building three separate multivariable models we find that none of the potential confounders altered the relationship between baseline S/CO, follow-up S/CO, or the change in S/CO and the joint PCR/ECG status. As such, only univariable association are reported.

**Table 3:** Results of univariable logistic regression models predicting joint PCR and ECG status at follow-up.

	OR(95%CI)	p-value
**Age (years)**	0.98 (0.96 to 1.01)	0.156
**Sex (male)**	0.82 (0.50 to 1.35)	0.437
**Hypertension**	1.10 (0.66 to 1.84)	0.710
**Diabetes**	1.63 (0.78 to 3.36)	0.189
**Smoking status** (current or previous)	0.58 (0.35 to 0.95)	0.033
**Donation S/CO <4**	6.89 (2.31 to 24.2)	0.001
**Follow-up S/CO <4**	4.85 (2.25 to 11.1)	<0.001
**Decline >1 S/CO**	5.40 (2.13 to 15.5)	0.001

Odds ratios (OR) greater than 1 indicate variables associated with joint negative PCR and normal ECG status at follow-up

## Discussion

We report a retrospective cohort of *T*. *cruzi* seropositive blood donors with longitudinal measurement of antibody levels over a median of 12 years follow-up. We have shown that the global humoral response to *T*. *cruzi* antigens–as measured by a highly-sensitive anti-*T*. *cruzi* ELISA–correlates with PCR positivity and electrocardiographic alterations. In particular, untreated individuals with spontaneously falling serology presented a lower proportion of positive PCR results and cardiomyopathy compared to those with other antibody trajectories. This is a novel finding, to the best of our knowledge.

The ECG alterations reported in this study are non-specific and highly prevalent in older adults without Chagas disease [22]. Therefore, a meaningful comparison group would be similarly aged seronegative blood donors. Ribeiro et al (2013) compared ECG findings in 500 seropositive and 500 seronegative blood donors in São Paulo and Montes Claros, Brazil [21]. The definition of major ECG alterations was broader than in the present study, including premature ventricular beats. Despite this, the overall prevalence of major alterations in the seropositive wing was lower (26%) than in our cohort (35%). This may reflect the older age (median 56 vs 48 years) and higher prevalence of hypertension (38% vs 23%) and diabetes (12% vs 5%) in our study. However, the prevalence of major alterations among seronegative donors was comparable to low-antibody participants (< 4 S/CO at follow-up) in our cohort: 9% and 11%, respectively.

Of the electrocardiographic abnormalities we evaluated, RBBB was most strongly associated with serology. In a recent systematic review of population-based studies (community surveys and blood donors) comparing healthy seropositive and seronegative individuals, RBBB was the most specific finding for Chagas disease [23]. The pooled prevalence of RBBB among *T*. *cruzi* infected individuals was 27%, compared to 5% among controls. In our cohort, the prevalence among low-antibody individuals was similar (3%), and in-line with seronegative arms in two comparable Brazilian cohorts [21,22].

Development of Chagasic cardiomyopathy is thought to be a direct consequence of parasite persistence within cardiac tissue [24], and parasitaemia, as detected by positive PCR, has been associated with the presence of typical ECG alterations among treated [25] and untreated [26] patients. Taken together, the observation that electrocardiographic findings among low-antibody individuals are similar to comparable seronegative populations supports the notion of spontaneous parasite clearance. The association between a negative antibody trend and ECG changes–albeit less pronounced than the absolute antibody level–also supports this hypothesis.

A number of other lines of evidence suggest that the intensity of the humoral response to *T*. *cruzi* infection is a function of ongoing parasite (antigenic) load. Long-term follow-up of patients treated with benznidazole has documented reductions in antibody levels, and occasional complete seroreversion, as measured by conventional [27] and multiplex serology [17,28–30]. Indeed, the consensus definition of treatment success is seroreversion on two of three conventional serologic tests. There are case reports of complete spontaneous seroreversion with clearly documented evidence of initial infection in patients with no apparent clinical disease [12,14]. However, in contrast to these lines of argument, rare case reports exist (for example [31]) of typical Chagasic ECG changes in patients with complete treatment-induced seroreversion, implying Chagas cardiomyopathy can develop following apparent treatment success. The significance of these findings is unclear as conduction abnormalities can develop during acute infection and may have preceded treatment initiation. Furthermore, as mentioned above, although ECG findings may be typical of Chagas disease (e.g. RBBB), they also occur in the setting of other cardiomyopathies, such as those due to hypertension and ischaemic heart disease.

In addition to these observations, we have previously shown [15], and now confirmed, that antibody levels measured at a single timepoint correlate with PCR positivity in patients with chronic CD. Furthermore, in this cohort 71% of subjects with low antibody levels (<4 S/CO) at follow-up had both a negative PCR and a normal ECG–both established proxies of parasite persistence. These findings suggest that, as a single measurement, a quantitative serology may be more informative than PCR, given the relatively stability of serology in the short- to medium-term and the issues of false-negatives with PCR.

In a sample of blood donors from the Argentinian Chaco province (20% *T*. *cruzi* seroprevalence), the distribution of antibody levels, as measured by a number of different assays, was clearly bimodal [8]. Although our sample size is small, restricting the conclusions that can be drawn, we do demonstrate apparently bimodal distributions of ELISA S/CO both at the time of donation and among jointly PCR and ECG negative subjects at both timepoints (Fig 1). PCR is frequently false-negative, whereas a positive result is unequivocal evidence of parasite persistence (excluding lab errors such as sample contamination). The ECG can also be normal in the context of parasite persistence–traditionally referred to as the indeterminate form of Chagas disease.

Therefore, the bimodal distribution of antibody levels in this PCR-ve and ECG normal group may be interpreted as follows. The population of donors in the low antibody peak represent individuals that have substantially reduced (or possibly completely cleared) the parasite. Their risk of developing ECG alterations would be the same as the non-Chagas population and PCR would be expected to be consistently negative on repeat testing. By contrast, the high antibody peak represents individuals with ongoing parasite load but false-negative PCR and normal ECG (as yet). This group might be expected to develop ECG alterations at a higher rate than the age-matched non-Chagas population and to have positive PCR if serial samples were taken.

### Limitations

The VITROS Anti-*T*. *cruzi* assay is semiquantitative. This means the S/CO value only approximately reflects the amount of anti-*T*. *cruzi* antibody in a given sample. Because this source of measurement error is unlikely to be related to the outcomes–i.e. it is a non-differential measurement bias–it will have biased towards the null hypothesis. Furthermore, in calculating the difference between donation and follow-up samples their measurement errors were combined. Therefore, the change overtime in S/CO is inherently noisier than a measurement at a single timepoint. This may partly explain the association between follow-up S/CO and ECG/PCR being more pronounced than the association with the change in S/CO overtime.

Furthermore, because the Vitrios Anti-*T cruzi* assay is based on whole-parasite antigens, we could not look for more refined associations with specific antibodies. For example, recent work in Bolivia and Peru has shown an association between seropositivity against a lineage specific (TcII/TcIV/TcVI) epitope and severity of cardiac disease [32], with similar results in Brazil [33]. This is a detail that needs to be further explored and could not be addressed in the present cohort.

The median age of this cohort was 56 years. Therefore, initial infection with *T*. *cruzi* was likely a distant event for most participants. We can infer this for two reasons. Firstly, São Paulo does not have active *T*. *cruzi* transmission and most participants were born in countryside endemic regions, having subsequently moved to São Paulo. Secondly, effective vectoral control over the last 30 years has greatly reduced the number of incident cases [11], with a clear cohort effect. This means that only individuals that remained seropositive into their 4^th^ to 7^th^ decade were included. As such, people achieving spontaneous parasite clearance early in the disease course–shortly following infection–and completely seroreverting by a younger age, would not have entered this study. In removing this group, our sampling frame may have resulted in a systematic underestimation of the rate of seroreversion.

Another limitation of this cohort was the storage of frozen donation samples for many years. This process will have artificially reduced the measured S/CO on these historical samples. Thus, the magnitude of seroreductions will have been systematically underestimated and any increase in antibody levels will have been overestimated. However, the alternative approach–comparing assay results performed many years apart–introduces variations in assay manufacture and protocol. This is probably an even more significant source of error.

## Conclusions

Our results, if further confirmed, suggest that quantitative serology based on whole cell lysate is an informative marker of parasite persistence and disease status in Chagas disease. The association between spontaneously falling antibody level, negative PCR and normal ECG support the notion of spontaneous clearance, but further evidence is needed. For example, we are evaluating more refined assays for characterization of antibody levels to specific *T cruzi* antigens using selected recombinant antigens in Luminex and protein array technologies. These assays will be evaluated on this and multiple other cohorts of untreated and treated clinical cases. These include longitudinal samples from younger seropositive donors from the Argentinian Chaco with PCR and clinical data. These ongoing studies, using the Ortho VITROS assay and more refined quantitative *T cruzi* antibody technologies, should provide further insights into the relationships between antibody dynamics and parasite persistence and clinical implications of spontaneous and treatment induced control or eradication of *T cruzi* infection.

## Supporting information

S1 Table Screening assays in routine use at the Fundação Pró-Sangue for the purpose of screening blood donations for *T*. *cruzi* infection. (DOCX)

S2 Table Comparison of ECG findings classified as major and typical of Chagas, and their distribution according to antibody level. X indicates that an ECG finding belongs to either the group of major or Chagas-typical findings, or both. Ψ RBBB + LAHB is included in the count of RBBB
(DOCX)

S3 Table Comparison of PCR result and ECG findings according to change in S/CO over time: decreasing, stable or increasing. (DOCX)

S4 Table Comparison of cardiovascular risk factors according to the absolute difference in VITROS S/CO between donation and follow-up and according to the S/CO value at follow-up. (DOCX)
