# Supplementary material for: Declining antibody levels to Trypanosoma cruzi correlate with polymerase chain reaction positivity and electrocardiographic changes in a retrospective cohort of untreated Brazilian blood donors
Source: PLoS Negl Trop Dis. 2020 Oct 27;14(10):e0008787. doi: 10.1371/journal.pntd.0008787 (PMC7647114; doi:10.1371/journal.pntd.0008787)
Supplement: S1 Table — (DOCX) [file pntd.0008787.s001.docx]

**S1 Table**

| **Period** | ***T. cruzi* screening test in use at FPS** |
| --- | --- |
| 1996 - 2002 | Considered positive if reactive by at least one of the following:  Hemacruzi BioLab Merieux – hemagglutination  ImunoCruzi BioLab Merieux – immunofluorescence  HBK 401 Hemobio Chagas - ELISA |
| 2003 - 2010 | BioMerieux – ELISA |
| 2011 | BioMerieux – ELISA (until 05/09/2011)  BioChile – ELISA (after 06/09/2011) |
| 2012 | BioChile – ELISA (until 09/10/2012)  Abbott - ELISA (after 10/10/2012) |
| 2013 - 2017 | Abbott - ELISA |
