# Supplementary material for: Declining antibody levels to Trypanosoma cruzi correlate with polymerase chain reaction positivity and electrocardiographic changes in a retrospective cohort of untreated Brazilian blood donors
Source: PLoS Negl Trop Dis. 2020 Oct 27;14(10):e0008787. doi: 10.1371/journal.pntd.0008787 (PMC7647114; doi:10.1371/journal.pntd.0008787)
Supplement: S2 Table — X indicates that an ECG finding belongs to either the group of major or Chagas-typical findings, or both. Ψ RBBB + LAHB is included in the count of RBBB (DOCX) [file pntd.0008787.s002.docx]

**S2 Table**

| ECG abnormalities | Major alterations | Typical alterations | Reduction in S/CO > 1  n = 56 | Reduction in S/CO ≤ 1  or increasing  n = 197 | Follow-up S/CO < 4  n = 35 | Follow-up S/CO ≥ 4  n = 241 | Donation S/CO < 4  n = 19 | Donation S/CO ≥ 4  n = 234 |
| --- | --- | --- | --- | --- | --- | --- | --- | --- |
| Major Q-wave abnormalities | X | X | 5 (8.9) | 22 (11.2) | 1 (2.9) | 30 (12.4) | 1 (5.2) | 26 (11.1) |
| Minor q-wave plus ST-T abnormalities | X | X | 1 (1.8) | 1 (0.5) | 1 (2.9) | 2 (8.3) | 0 (0.0) | 2 (1.0) |
| Major isolated ST-T abnormalities | X | X | 3 (5.4) | 21 (10.7) | 3 (8.6) | 25 (10.4) | 1 (5.3) | 23 (9.8) |
| Major Q-wave prolongation | X |  | 2 (3.6) | 11 (5.6) | 1 (2.9) | 14 (5.8) | 0 (0) | 13 (5.6) |
| LBBB | X | X | 0 (0.0) | 3 (1.5) | 0 (0) | 4 (1.7) | 0 (0) | 3 (1.3) |
| RBBB ^Ψ^ | X | X | 6 (10.7) | 40 (20.3) | 1 (2.9) | 48 (19.9) | 0 (0) | 46 (20.0) |
| RBBB + LAHB | X | X | 1 (1.8) | 6 (3.0) | 0 (0) | 8 (3.3) | 0 (0) | 7 (3.0) |
| Intraventricular blocks | X | X | 0 (0) | 1 (0.5) | 0 (0) | 3 (1.2) | 0 (0) | 1 (<1) |
| Atrial fibrillation/flutter | X | X | 1 (1.8) | 4 (2.0) | 0 (0) | 7 (3.0) | 0 (0) | 5 (2.1) |
| Pace maker | X | X | 0 (0) | 2 (1.0) | 0 (0) | 3 (1.2) | 0 (0) | 2 (1.0) |
| 2^nd^ degree AV block | X | X | 0 | 1 (0.5) | 0 (0) | 1 (0.4) | 0 (0) | 1 (<1) |
| 3^rd^ degree AV block | X | X | 0 (0) | 0 (0) | 0 (0) | 0 (0) | 0 (0) | 0 (0) |
| Left ventricular hypertrophy plus ST-T abnormalities | X |  | 0 (0) | 2 (1.0) | 0 (0.0) | 4 (1.7) | 0 (0) | 2 (1.0) |
| Supraventricular tachycardia | X |  | 0 (0) | 0 (0) | 0 (0) | 0 (0) | 0 (0) | 0 (0) |
| Ventricular pre-excitation (WPW | X |  | 0 (0) | 0 (0) | 0 (0) | 0 (0) | 0 (0) | 0 (0) |
| Sinus bradycardia |  | X | 0 (0) | 0 (0) | 0 (0) | 0 (0) | 0 (0) | 0 (0) |
