# Supplementary material for: Declining antibody levels to Trypanosoma cruzi correlate with polymerase chain reaction positivity and electrocardiographic changes in a retrospective cohort of untreated Brazilian blood donors
Source: PLoS Negl Trop Dis. 2020 Oct 27;14(10):e0008787. doi: 10.1371/journal.pntd.0008787 (PMC7647114; doi:10.1371/journal.pntd.0008787)
Supplement: S3 Table — (DOCX) [file pntd.0008787.s003.docx]

**S3 Table**

|  | **Difference in S/CO (follow-up - donation)** | | |
| --- | --- | --- | --- |
|  | **< -1 S/CO units**  N = 56  n (%) or mean (sd) | **>= -1 and < +1 S/CO units**  N = 126  n (%) or mean (sd) | **>= +1 S/CO units**  N= 17  n (%) or mean (sd) |
| **PCR result**  Positive  Negative | 15 (26.8)  41 (73.2) | 66 (52.4)  60 (47.6) | 38 (53.5)  33 (46.5) |
| **Final ECG classification**  Major  Minor  Normal | 11 (19.6)  29 (51.8)  16 (28.6) | 46 (36.5)  56 (44.4)  24 (19.1) | 30 (42.3)  26 (36.6)  15 (21.1) |
| **PCR negative + ECG normal or minor alteration** | 36 (64.3) | 42 (33.3) | 20 (28.2) |
| **Typical changes,**  0  1  2+ | 46 (82.1)  6 (10.7)  4 (7.1) | 80 (63.5)  39 (31.0)  7 (5.5) | 42 (59.2)  18 (25.3)  11 (15.5) |
| **No. typical changes per trace** | 0.27 (0.65) | 0.43 (0.63) | 0.56 (0.75) |
