# Supplementary material for: Declining antibody levels to Trypanosoma cruzi correlate with polymerase chain reaction positivity and electrocardiographic changes in a retrospective cohort of untreated Brazilian blood donors
Source: PLoS Negl Trop Dis. 2020 Oct 27;14(10):e0008787. doi: 10.1371/journal.pntd.0008787 (PMC7647114; doi:10.1371/journal.pntd.0008787)
Supplement: S4 Table — (DOCX) [file pntd.0008787.s004.docx]

**S4 Table**

| Subject characteristics | Reduction in S/CO > 1  n = 56 | Reduction in S/CO ≤ 1  or increasing  n = 197 | Follow-up S/CO < 4  n = 35 | Follow-up S/CO ≥ 4  n = 241 | Donation S/CO < 4  n = 19 | Donation S/CO ≥ 4  n = 234 |
| --- | --- | --- | --- | --- | --- | --- |
| Age (years), n (%)  < 40  40 – 49  50 – 59  60 – 69  >= 70 | 1 (1.8)  14 (25.5)  19 (34.5)  17 (30.9)  4 (7.3) | 14 (7.1)  38 (19.3)  71 (36.0)  57 (28.9)  17 (8.6) | 2 (5.9)  7 (20.6)  11 (32.4)  11 (32.4)  3 (8.8) | 13 (5.4)  49 (20.4)  85 (34.4)  71 (29.6)  22 (9.2) | 2 (10.5)  5 (26.3)  8 (42.1)  3 (15.8)  1 (5.3) | 13 (5.6)  47 (20.1)  83 (35.5)  71 (30.3)  20 (8.5) |
| Sex, n(%)  Male  Female | 24 (42.8)  32 (57.2) | 98 (49.7)  99 (50.3) | 14 (40.0)  21 (60.0) | 119 (49.4)  122 (50.6) | 8 (42.1)  11 (57.9) | 114 (48.7)  120 (51.3) |
| Smoking status, n (%)  Current smoker  Ex-smoker  Never smoker | 5 (8.9)  20 (35.7)  31 (55.4) | 18 (9.1)  78 (39.6)  101 (51.3) | 6 (18.2)  10 (27.3)  19 (54.5) | 18 (7.5)  98 (40.7)  125 (51.9) | 5 (26.3)  2 (10.5)  12 (63.2) | 18 (7.7)  96 (41.0)  120 (51.3) |
| Comorbidities, n (%)  Diabetes  Hypertension  Dyslipidemia | 7 (12.7)  26 (48.1)  16 (29.1) | 23 (11.9)  68 (34.7)  68 (37.8) | 3 (8.8)  15 (44.1)  11 (31.4) | 31 (13.1)  88 (37.1)  83 (37.4) | 2 (10.5)  8 (44.4)  7 (36.8) | 28 (12.2)  86 (37.1)  77 (35.6) |
